# A new species of *Clinanthus* from northern Peru (Asparagales, Amaryllidaceae, Amarylloideae, Clinantheae)

**DOI:** 10.3897/phytokeys.63.8895

**Published:** 2016-06-02

**Authors:** Segundo Leiva, Alan W. Meerow

**Affiliations:** 1 Universidad Privada Antenor Orrego, Av. América Sur 3145 Casilla postal 1075, Trujillo, Perú; 2 USDA-ARS-SHRS, National Germplasm Repository, 13601 Old Cutler Road, Miami, Florida 33158, USA

**Keywords:** Geophyte, Andes, Clinantheae, new species, taxonomy

## Abstract

*Clinanthus milagroanthus* S. Leiva & Meerow, **sp. nov.** is described from the Department of La Libertad in Peru. The new species is most closely related to *Clinanthus mirabilis* (Ravenna) Meerow, with further affinities to *Clinanthus viridiflorus* (R. & P.) Meerow. It can be distinguished from *Clinanthus mirabilis* by its wider leaves, the much more brightly colored and wide spreading limb, and the much lighter colored perigone tube (yellowish green vs. dark green in *Clinanthus mirabilis*). A conspicuous bulge just proximal to the midpoint of the tube is a unique character of the new species.

## Abstract (es)

*Clinanthus milagroanthus* S. Leiva & Meerow, **sp. nov.** se describe desde el Departamento de La Libertad, Perú. La nueva especie es más estrechamente relacionada con *Clinanthus mirabilis* (Ravenna) Meerow, con adicionales afinidades con *Clinanthus viridiflorus* (R. & P.) Meerow. Se puede distinguir de *Clinanthus mirabilis* por sus hojas más anchas, el limbo mucho más amplio de colores brillantes, y el color de tubo perigonio mucho más claro (color amarillento vs. verde verde oscuro en *Clinanthus mirabilis*). Una protuberancia visible casi proximal al punto medio del tubo es un carácter único de la nueva especie.

## Introduction

Peru is the center of diversity for the genus *Clinanthus* Herb., which was segregated from *Stenomesson* Herb. by Meerow et al. (2000), who demonstrated that the latter was polyphyletic. There are between 15 and 20 species in the genus, which has never been monographed. The species are primarily known from locations above 2000 m (León et al. 2013; unpubl. herbarium data), but a cluster of species has colonized the Peru Current-cooled *lomas* of the coast (León et al. 2013; unpubl. herbarium data). One species, *Clitanthes humilis* (Herb.) Meerow, which retains the ovary inside the bulb until shortly before seed ripening (Herbert 1839; Baker 1871), reaches elevations above 4000 m (León et al. 2013; unpubl. herbarium data). Many are local endemics known only from the type localities (León et al. 2013). Exploration in the Department of La Libertad has uncovered a species new to science, and it is herein described.

## Materials and methods

No specimens matching the new species have been observed in herbarium collections in Peru, nor encountered by the second author in collections examined over the past 30 years at GB, K, MO, and NY. The description is based upon live material from the type collection. Colors are referenced to the Royal Horticultural Society (RHS) Color Charts (RHS 1995).

## Taxonomy

### Clinanthus milagroanthus

Taxon classificationPlantaeAsparagalesAmaryllidaceae

S. Leiva & Meerow sp. nov.

urn:lsid:ipni.org:names:77155329-1

Figs 1 , 2

#### Diagnosis.

*Clinanthus milagroanthus* is most closely related to *Clinanthus mirabilis* (Ravenna) Meerow (Fig. 3A) by the white color and morphology of the staminal cup, in which the free portions of the filaments are slightly incurved and inserted at the sinus between the cup lobes. Both of these species have affinity with *Clinanthus viridiflorus* (R. & P.) Meerow (Fig. 3B–C). All three species have grayish-green glaucous leaves, particularly large apicula at the apex of the outer tepals, and large anthers relative to other species of the genus. These species form a distinct clade in the genus based on ribosomal DNA sequences with close relationship to the genus *Paramongaia* Velarde (Fig. 3D; Meerow et al. 2000; Ravenna 1988; unpubl. data). All three species have glaucous, gray-green leaves. The perigone of *Clinanthus viridiflorus* is entirely green (Fig. 3B–C); that of *Clinanthus mirabilis* is deep green except for the orange-red limb (Fig. 3A). Our new species has the widest leaves in the complex and bears the showiest flowers, most notably by the sharp constrast between the white staminal cup and the bright red tepals. It can be distinguished from *Clinanthus mirabilis*, to which it bears closest resemblance, by the much more brightly colored and wide spreading limb, and the much lighter colored perigone tube (yellowish green vs. dark green in *Clinanthus mirabilis*). The conspicuous bulge just proximal to the midpoint of the tube is a unique character of the new species.

**Figure 1.**
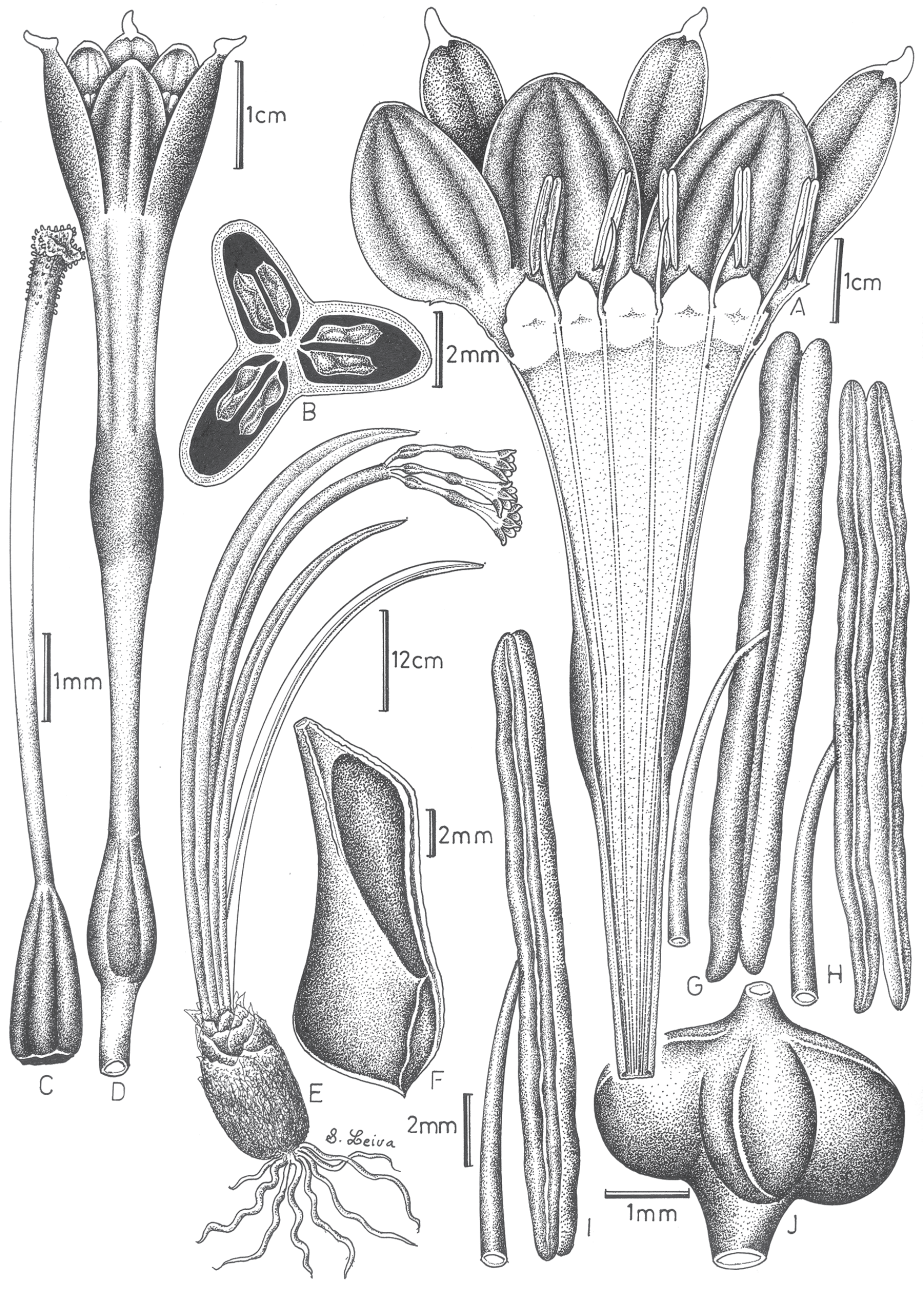
*Clinanthus milagroanthus* S. Leiva & Meerow. **A** Perigone dissected showing androecium **B** Cross-section of the capsule **C** Gynoecium **D** Flower at anthesis **E** Habit **F** Seed **G** Anther in dorsal view **H** Anther in ventral view **I** Anther in side view **J** Capsule. Drawing by S. Leiva & M. Leiva from S. & M. Leiva Leiva 5795 (HAO).

**Figure 2.**
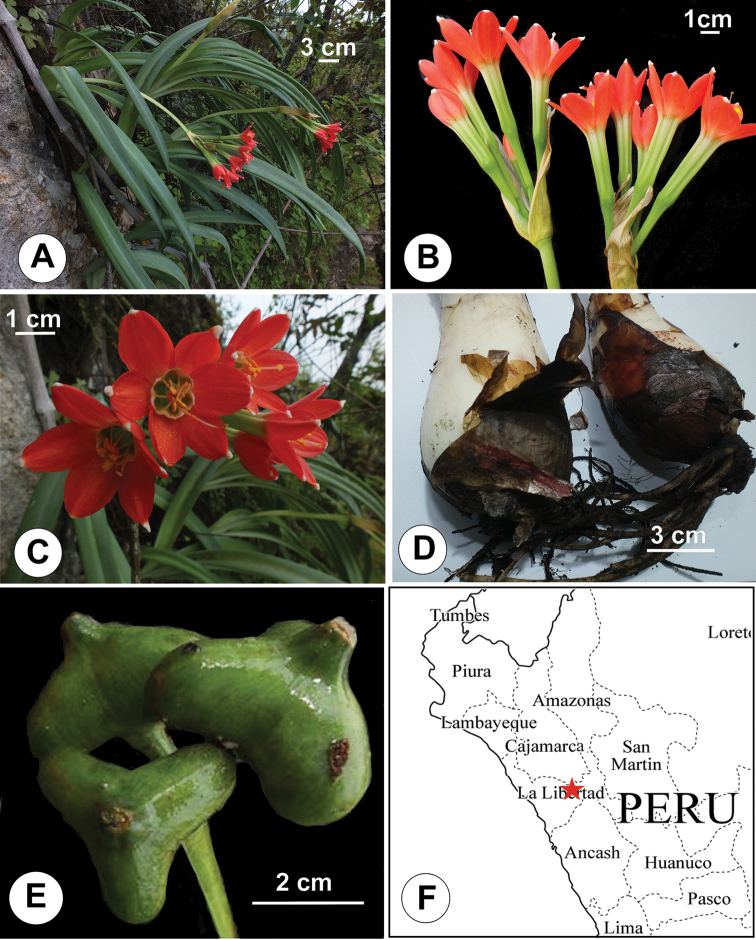
*Clinanthus milagroanthus* S. Leiva & Meerow. **A** Habit **B** Inflorescence showing spathe bracts **C** Flowers **D** Bulbs **E** Capsules **F** Known distribution of *Clinanthus milagroanthus* in Peru (red star). All photos of S. Leiva & M. Leiva 5795, HAO.

**Figure 3.**
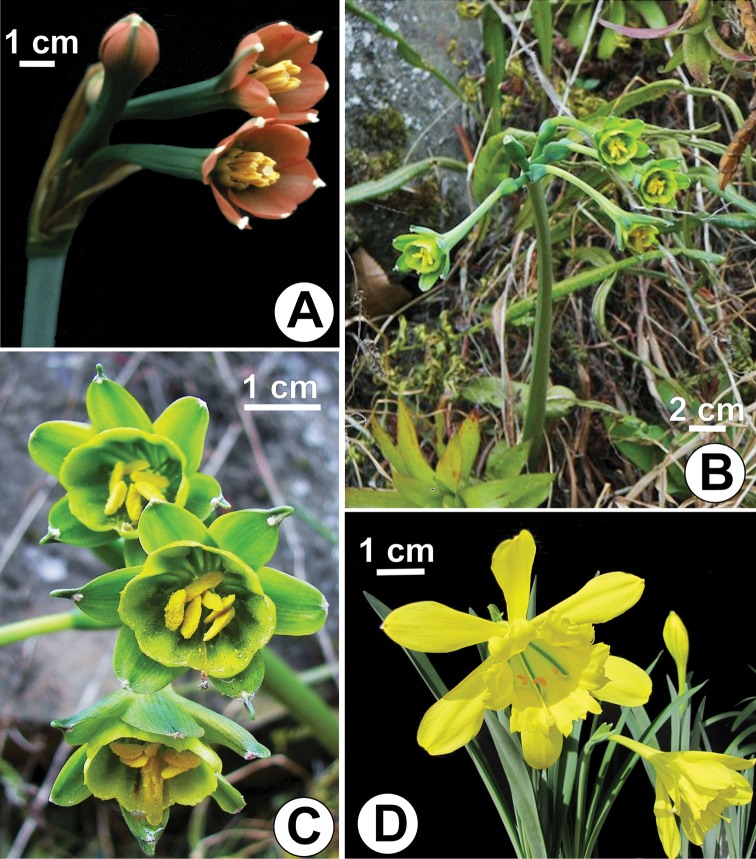
Species related to *Clinanthus milagroanthus*. **A** *Clinanthus mirabilis* (S. Leiva et al. 2000, HAO) **B–C** *Clinanthus viridiflorus* (Weigend et al. 5701, NY) **D** *Paramongaia weberbaueri* (Meerow 2303, FTG).

#### Type.

PERÚ. Dpto. La Libertad, Prov. Otuzco, Distrito Salpo, above Murañe (on the Salpo-Pagash road), 8°1'16.5"S, 78°33'16.2"W, 2827 m elevation, 22 Mar 2015, S. Leiva & M. Leiva 5795 (HOLOTYPE: HAO; ISOTYPE: F)

#### Description.

Geophytic herb from tunicate bulbs, 60–80 cm tall, with numerous creamy-white roots (brown where stained by the humic substrate), 25–35 cm long. Bulbs long conical or tapered, 9–10 cm long and 6.5–7 cm in diameter, with a papery brown to black tunic, white below. Leaves distichous, sessile, lorate, erect or slightly reflexed towards the abaxial surface, succulent, dark green on adaxial surface, light green on abaxial, glabrous, acute at apex, slightly cuneate at the base, slightly revolute at the margins, caniculate along the midrib adaxially, midrib prominent abaxially, (57-) 65–68 cm long by 5.5–5.7 cm wide. Inflorescences with 5 flowers arranged in pseudoumbels, scape ancipitous, elliptical in cross section, dull yellowish-green, succulent, solid, 35–36 cm long by 1.4–1.5 cm diameter; spathe bracts membranous, two, creamy or slightly yellowish, glabrous, eventually marcescent, surrounding the base of the flowers; pedicels slightly wider proximally, yellowish-green, succulent, glabrous, 2-edged, slightly curved towards the abaxial surface, 1–1.3 cm long by 0.4–0.5 cm in diameter. Flowers actinomorphic, bisexual, trimerous; perigone infundibular, succulent, 7–7.2 cm long, the tepals fused into a tube for 2/3 to 4/5 of the perigone length; tube RHS Green 143C green proximally, darkening in the inflated portion, then becoming Yellow-Green 145B to almost white at the throat, cylindrical for the proximal 1.2–1.5 cm and 2.5–3 mm diam., gradually dilated to 5 mm for the next 1–1.3 cm, then abruptly inflated to ca. 7 mm diam for ca. 1.3 cm, constricting distally to ca. 5 mm diam, the final 2 cm portion of the tube with 6 longitudinal channels; limb of six tepals in two series, spreading to 3.4–4.0 cm at anthesis at ca. 45°angle from the throat; outer tepals narrowly oblong, intensely red (RHS Red Group 40A to 40B) on both surfaces, with a conspicuous white, papillate apiculum at apex, glabrous on both surfaces, succulent, distinctly ribbed, 2.5–2.6 cm long by 1–1.2 cm wide; inner tepals broadly elliptic, same color and surface attributes as outer, obtuse at apex with a minute white apiculum, 2.2–2.3 cm long by 1.4–1.5 cm wide. Stamens six, connate proximally into a short, externally white 6-lobed staminal corona, stained yellowish-green internally towards the base, 5–6 mm long and 1.3–1.4 cm diam, the lobes deltoid with a mucronate apex, each 5–6 mm long and 5–6 mm wide; free portion of stamens filiform, inserted at the sinus between each lobe of the corona, incurved, white, 8–9 mm long; anthers narrowly oblong, sagittate at the base, introrse, 17–18 mm long and 2–2.1 mm wide, pollen bright yellow. Style exserted, filiform, creamy white, with translucent papillae distally, 80–83 mm long; stigma 3-lobed, white to slightly yellow, 1.5–1.6 mm wide. Ovary inferior, turbinate, 3-locular, 15–18 mm long, 6–7 mm wide; ovules numerous, superposed in two vertical rows in each locule. Capsule tricoccous, green when young, becoming glaucous with age, 2.4–2.6 cm high 3.5–4 cm wide, loculicidally dehiscent; seeds 100–105, flattened, slightly polyhedral, narrowly winged on the edges, covered with a lustrous, brittle, black phytomelanous testa, 17 to 18 mm long, 7 to 7.3 mm wide.

#### Distribution and ecology.

*Clinanthus milagroanthus* is only known from the local area of the type collection where it is moderately abundant. Despite having searched the surrounding area, it has so far been limited to the area of Muräne along the Salpo-Pagash road in the Department of La Libertad, Prov. Otuzco, District Salpo, ca. 8°01'16.4"S and 78°33'16.2"W, at 2824 m elevation, as a member of the grass and shrub vegetation on the edges of the road, preferring moist, black organic soil among rocks. Some associated species include *Escallonia micrantha* Mattf. (Escalloniaceae), *Bidens triplinervia* Kunth (Asteraceae), Austrocyndropuntia subulata subsp. exaltata (A. Berger) D. R. Hunt (Cactaceae), *Vicia andicola* Kunth (Fabaceae), *Puya casmichensis* L. B. Sm. (Bromeliaceae), *Begonia geraniifolia* Hook. (Begoniaceae), *Passiflora peduncularis* Cav. (Passifloraceae), and unidentifed *Smallanthu*s Mack., *Verbesina* L. (Asteraceae), *Solanum* L. (Solanaceae), *Lupinus* L. (Fabaceae). *Clinanthus milagroanthus* flowers with the first rains in November or December, continuing through fruit maturation until March or April.

#### Current conservation status.

Using the criteria of the IUCN (IUCN 2012) *Clinanthus milagroanthus* is considered critically endangered (CR). The extent of its range is less than 100 km^2^ around the type locality (Criterion B1), is known from a single population (Criterion B1a) with less than 100 mature individuals in the population, and is projected to decline further (Criterion B1b). It may have been impacted by construction of the road between Salpo to Pagash or Platanar. However, there has not been an assessment of whether it experienced a decline in its range from this event. The species requires a thorough study of ecology, population structure and distribution to clarify its status.

#### Vernacular name.

“cebolla de peña.”

#### Etymology.

The specific epithet honors Ms. Milagros Leiva Salinas, a student of Human Medicine, who has been studying the phytochemistry of Peruvian genera of Amaryllidaceae.

#### Additional material examined.

PERÚ. Dpto. La Libertad, Prov. Otuzco, Dist. Salpo, arriba del Murañe (ruta Salpo-Pagash), 8°01'16.4"S and 78°33'16.2"W, 2824 m, 1 Apr 2013, *S. Leiva & M. Leiva 5443* (HAO)

#### Notes.

We have used the original orthography for the names cited below. The genus *Callithauma* Herbert was established by Herbert (1837) to accommodate *Callithauma viridiflorum* (R. & P.) Herbert. He later (Herbert 1841) described a second sp., *Callithauma angustifolium*, which he distinguished by its narrower leaves, smaller flowers, exserted style and 3-lobed stigma. Bentham and Hooker (1883) subsumed the genus under *Stenomesson*. Baker (1878) described Callithauma viridiflorum var. elwesii, which Macbride (1931) raised to the rank of species in *Stenomesson*. Baker (1888) appeared to reduce Herbert’s (1841) *Callithauma angustifolium* to a variety of *Stenomesson viridiflorum*. Ravenna (1971) described *Stenomesson mirabile* and assigned it to subgenus *Callithauma* (but didn’t formally validate the new subgenus). He later (Ravenna 1988) restablished the genus *Callithauma*, with only *Callithauma viridiflorum*, noting its relationship to *Paramongaia*, and erected a new genus, *Anax* Ravenna, with *Anax elwesii* (Baker) Ravenna as the type species, and including *Anax mirabilis* (Ravenna) Ravenna. Ravenna’s (1988) diagnostic characters for distinguishing these two genera were somewhat ambiguous. Meerow et al. (2000) showed clear molecular evidence that the genus *Stenomesson* was polyphyletic, and established the new tribe Clinantheae for the genera *Clinanthus*, *Pamianthe* Sealy and *Paramongaia*. *Paramongaia weberbaueri* Velarde and *Clinanthus mirabilis* were resolved as sister species, in a clade in turn sister to three other species of *Clinanthus*. We are in the process and obtaining a larger number of DNA sequences of the tribe Clinantheae to better evaluate the generic limits within the tribe.

## Supplementary Material

XML Treatment for Clinanthus milagroanthus
